# Physician Perspectives on Responding to Clinician-Perpetuated Interpersonal Racism Against Black Patients with Serious Illness

**DOI:** 10.1007/s11606-023-08377-z

**Published:** 2023-08-24

**Authors:** Crystal E. Brown, Cyndy R. Snyder, Arisa R. Marshall, Kristine L. Cueva, Sandra Y. Jackson, Kemi M. Doll, Sherita H. Golden, Bessie Young, Abby R. Rosenberg

**Affiliations:** 1 Cambia Palliative Care Center of Excellence at UW Medicine, Seattle, WA USA; 2 Division of Pulmonary, Critical Care, and Sleep Medicine, Department of Medicine, University of Washington School of Medicine, Seattle, WA USA; 3 Department of Bioethics and Humanities, School of Medicine, University of Washington, Seattle, WA USA; 4 Department of Family Medicine, Center for Health Workforce Studies, School of Medicine, University of Washington, Seattle, WA USA; 5 Department of Medicine, University of Washington, Seattle, WA USA; 6 United States Army, Center for Army Analysis, Fort Belvoir, VA USA; 7 Division of Gynecologic Oncology, Department of Obstetrics and Gynecology, University of Washington, Seattle, WA USA; 8 Division of Endocrinology, Diabetes, and Metabolism, John Hopkins University, Baltimore, MD USA; 9 Division of Nephrology, Department of Medicine, University of Washington, Seattle, WA USA; 10 Department of Psychosocial Oncology and Palliative Care, Dana Farber Cancer Institute, Boston, MA USA; 11 Department of Pediatrics, Harvard Medical School, Boston, MA USA

## Abstract

**Background:**

Racism negatively affects clinical outcomes in Black patients, but uncertainty remains among physicians regarding how to address interpersonal anti-Black racism incidences involving them to facilitate racial healing and promote accountability.

**Objective:**

Elicit physician perspectives on addressing concerns from Black patients about interpersonal racism involving them or their team.

**Participants:**

Twenty-one physician subspecialists at an urban academic medical center.

**Approach:**

We conducted one-on-one semi-structured interviews to help inform the development of a clinician-facing component of a program to address the distress of racism experienced by Black patients with serious illness. We asked clinicians to describe experiences discussing racism with patients and identify additional resources to support these conversations.

**Main Measures:**

Physician perspectives, including barriers and facilitators, to promote racial healing and clinician accountability when discussing clinician-perpetuated interpersonal racism with Black patients.

**Key Results:**

Of the 21 participating physicians, 67% were women with a mean age of 44.2 years and mean of 10.8 years of experience as an attending physician. Four identified as Asian, three identified as Black, and 14 identified as White. Participants largely felt unprepared to discuss racism with their patients, especially if the harm was caused by them or their team. Participants felt patients should be given tools to discuss concerns about racism with their clinicians, but worried about adding additional burdens to Black patients to call out racism. Participants believed programs and processes with both patient- and clinicians-facing components had the potential to empower patients while providing resources and tools for clinicians to engage in these highly sensitive discussions without perpetuating more harm.

**Conclusions:**

Addressing and improving communication about interpersonal racism in clinical settings are challenging. Dual-facing programs involving patients and clinicians may help provide additional resources to address experiences of interpersonal racism and hold clinicians accountable.

**Supplementary Information:**

The online version contains supplementary material available at 10.1007/s11606-023-08377-z.

## INTRODUCTION

Anti-Black racism and structural injustices fuel health and healthcare inequities that systematically disadvantage Black patients, diminish their well-being, and contribute to poor outcomes.^1^ As a result, Black patients experience many of the known negative effects of racism including worse health outcomes and increased mortality.^2–5^ This is true even during serious illness as Black patients approach end-of-life (EOL). In the last 6 months of life, Black patients are more likely to experience poor-quality communication, higher intensity care, increased financial strain, and high symptom burden resulting in poor quality of life.^6,7^ These inequities are reflective of structural and interpersonal racism Black patients experience both outside of and within the clinical setting.^6^ While structural racism refers to material conditions, power, and privilege within healthcare institutions, interpersonal racism refers to a healthcare worker’s (HCW) discriminatory and prejudiced acts, whether intentional or unintentional, that results in differential racial treatment of patients.^8^

In a related study, we highlighted how Black patients with serious illness experienced epistemic injustice. Epistemic injustice is the discrediting of beliefs and knowledge of another person, often due to their social identity.^9^ Patients described the ongoing silencing and smothering of their own knowledge and lived experiences by HCWs as the most common manifestation of anti-Black racism in the clinical setting.^10^ Patients also reported high levels of race-based mistrust and discriminatory treatment from HCWs in accompanying surveys using validated measures of mistrust, microaggressions, and discrimination in clinical settings.^10–13^ During semi-structured interviews, patients described placating behaviors and general lack of receptivity from HCWs as a significant barrier to racial healing and repairing the harms of anti-Black racism. As a result, HCWs rarely took accountability for their actions or the harm they perpetrated upon Black patients. These harms were compounded by additional intersecting axes of oppression, such as perceived ability to pay and homelessness, further impacting the lived experiences of Black patients. These data suggest that physicians may feel defensive or uncomfortable with discussing Black patients’ concerns with racism, especially if it involves them or members of their team.^14^ While an increase in trainings in equity and diversity includes efforts to recognize the impact of racism on clinical outcomes, it is unclear if it encourages physicians and other HCWs to bring anti-racist, inclusive approaches to their care or to promote accountability when HCWs have harmed Black patients.

In this study, we conducted semi-structured interviews with physicians experienced with caring for patients with serious illness to elicit physician skills and resources needed to improve willingness and self-efficacy to discuss racism concerns, particularly if it involved them or members of their team. Our goal was to identify additional resources for clinicians to navigate these difficult, racially charged conversations to foster racial healing and promote clinician accountability.

## METHODS

### Study Design

This is a qualitative study of semi-structured interviews using thematic analysis to elicit barriers identified by physicians to discussing racism concerns during clinical care, particularly if it involved them or members of their team, and identify resources to improve willingness and self-efficacy to discuss them and take accountability for their harms. We used thematic analysis to identify patterns and themes that emerged iteratively from our data.^15^ This was done to aid the development of a clinician-facing component of a dual-facing program (“Promoting Resources in Stress Management” (PRISM)) to manage the distress of racism among Black patients. PRISM is a coach-led program initially designed to improve personal resilience resources.^16,17^ Personal resilience resources are necessary to respond to the distress of racism,^18–20^ but modern resilience concepts can perpetuate bias, stigma, and deficit-based approaches to marginalized communities. Current concepts of resilience do not account for disproportionate socioeconomic disadvantage, unequal power dynamics, discrimination, and other inequities experienced by Black and other racially marginalized communities.^18,21–23^ For example, structural inequities result in environments in which marginalized populations must disproportionately use personal resilience resources to navigate injustices, but these structural inequities are rarely acknowledged. Discourse around resilience focuses more on marginalized individuals and the interventions targeting them rather than the social or political climates that requires them to be more resilient than others.

For our purposes, PRISM was modified, based on feedback from Black patients with serious illness (Supplemental Table 1), to directly encourage discussions on racism, provide support and validation, and facilitate racial healing during admissions and other healthcare interactions. This feedback included learning additional stress management skills, improving knowledge about the inner workings of hospitals and team structures, and providing tools and resources to raise concerns about anti-Black racism in their care. Patients reported that a clinician-facing component of PRISM was necessary to discuss prior or current experiences with racism and facilitate accountability, decrease distress, and promote racial healing. Hence, we wanted to explore physician willingness and self-efficacy to discuss racism and take accountability for its harms, while exploring how a dual-facing version of PRISM that is deployed in the clinical setting might help.

### Participants

We conducted one-on-one semi-structured interviews with medical subspecialists at an urban academic county hospital at the University of Washington. Physicians were purposefully identified from Division websites to capture a diversity of characteristics including academic rank, race, gender, and specialty. Potential participants were emailed a study description and information sheet. Interviews were conducted virtually between August and October 2022. A $20 gift card was provided in appreciation of their participation. This study was deemed exempt by the University of Washington Institutional Review Board (STUDY00016935).

### Interview Guide

The interview guide included a description of PRISM and open-ended questions regarding the program and racism. The guide was adapted from a related study examining experiences with racism among Black patients with serious illness (Supplemental Material). After an overview of the PRISM program, participants were asked to provide their general thoughts. Next, participants were asked about prior experiences discussing anti-Black racism with patients with serious illness. Participants were provided examples of patient concerns around anti-Black racism in their care to help facilitate discussions. These examples were drawn from a related qualitative study on racism experiences of Black patients with serious illness. Participants were asked whether they had experienced similar interactions, and what resources or tools may help them address patients’ concerns about anti-Black racism with a focus toward accountability, especially if they or their team were the offending clinicians.

### Analysis

We describe researcher positionality as beliefs, understandings, and lived experiences as it relates to race and racism shapes analyses and interpetation.^15,24^ CEB, a biracial Black and Korean pulmonary and critical care physician trained in mixed methods research, conducted all interviews. CEB had prior professional, working relationships with nine of the participants. Interviews were individual and 30–60 min long. Interviews were conducted beyond thematic saturation.^15^ Thematic saturation was defined as the point when no new additional unique patterns or themes emerged from interviews.^25^ All interviews were recorded, deidentified, and transcribed.

The research team performed inductive coding of the transcribed manuscripts. CEB and CRS, a multiracial Black qualitative researcher with experience in health equity research, reviewed the transcripts and produced a list of themes, subthemes, and codes. The preliminary themes and codes were reviewed by the research team including ARM, a mixed race, Asian and Pacific-Islander research coordinator with experience in healthcare ethics, and KLC, a Filipina American internal medicine resident. The team independently coded an initial set of three transcripts, then compared codes, identifying similarities and differences. We reconciled discrepancies through consensus, refined codes, and established definitions. CEB, KLC, and ARM coded and co-reviewed the remaining 18 transcripts. DeDoose (www.dedoose.com) was used to support the analyses.

## RESULTS

**Figure 1:**
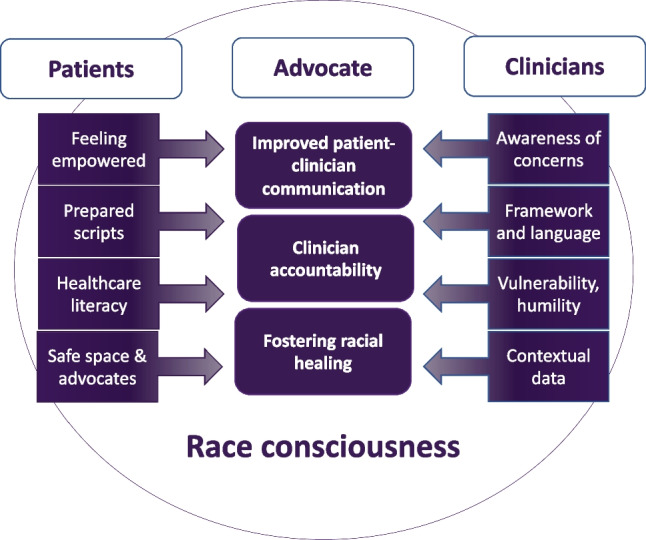
Conceptual model of the findings in our study. This figure depicts additional skillsets and support identified by study participants to help foster productive conversations between patients and clinicians to improve communication, foster racial healing, and promote accountability. Race consciousness draws attention to racial dynamics within a racialized medical system.

Twenty-one physicians enrolled in the study with a participation rate of 60% (Table 1). Participants were mostly women (67%). Four identified as Asian, and three identified as Black. All participants agreed that discussing patient concerns about racism was important. Participants responded positively that PRISM was patient-focused with clear objectives and widely applicable content. One physician noted, “These are very important tools for any issue. I wish somebody taught me this in medical school.” We identified three broad themes around processing and responding to patient concerns regarding anti-Black racism: (1) receiving and processing difficult-to-hear feedback, (2) clinician skills and resources to foster productive discussions about racism, and (3) supporting patients through difficult conversations (Fig. 1). Potential solutions were discussed with participants (Table 2).

**Table 1:** Participant Characteristics (*N* = 21)

Variable	Statistic
Age*	44.2 (7.8)
Female^†^	14 (66.7)
Years in practice*	10.8 (7.4)
Self-reported race	
White	14 (71.4)
Black	3 (14.3)
Asian	4 (19.0)
Specialty^†^	
Pulmonary and critical care	8 (38.0)
Nephrology	5 (23.8)
Infectious disease	3 (14.3)
Gastroenterology	3 (14.3)
Cardiology	2 (14.3)
Rank^†^	
Assistant professor	9 (28.6)
Associate professor	6 (28.6)
Professor	4 (19.0)

^*^Mean (SD); ^†^*N* (%)

**Table 2:** Physician-Identified Barriers and Proposed Resources and Skills to Address Interpersonal Anti-Black Racism

Barrier	Solution	Example of potential solutions
Clinician barriers		
Unaware or appeasement of concerns	Awareness of concerns	Racism concerns brought to clinician’s attention either by patient or patient advocate
Lack of guidance or language	Framework and language	Learn about or access frameworks as well as “do’s and don’ts” when discussing racism with patients
Authoritarian or paternalistic practices	Vulnerability and humility	Defensiveness is processed away from the patient and discussed with colleagues or team. Humility-based listening is discussed in preparation for conversations with patient
Lack of data or evidence	Contextual data	Contextual data provided showing known, documented effects of racism in health and healthcare outcomes
Patient barriers		
Unequal power dynamics	Feeling empowered	Provided with additional knowledge about hospital or medical culture to optimize approach
Fear of speaking up	Prepared scripts	Prepares concerns with empowering language with advocate. Patient provided with “low stakes,” back-of-pocket phrases when concerns about racism arise
Unsafe environment	Healthcare literacy	Encouraged to ask questions about their medical care and the medical system in presence of advocate or trusted clinician; hospital systems hire more Black and brown physicians
Marginalized intersecting identities	Safe space and advocates	Patients feel heard and their intersecting identities recognized through resources including their support system, patient advocates, available peer support groups

### Receiving and Processing Information

#### Transparency

All participants welcomed the opportunity to hear patients’ concerns about racism. One physician wondered if patients were hesitant to discuss racism with him because of his race: “I worry as a White guy, do people not want to talk to me about it?” Participants thought it was important to be aware of these concerns and address them, even if it made them uncomfortable: “If I was causing distress on any marginalized patient in a manner they felt was due to their race, I want to know.” Participants wanted to identify and eliminate seemingly innocuous behavior that actually harms patients: “I like the idea of being able to give feedback and facilitate discussions so we don’t continue on with adverse practices.”

#### Finding Time and Space

Participants thought it was important that patients’ concerns are delivered without the patient present. Many participants initially wanted to hear about the concerns from the PRISM coach since many were worried about feeling defensive when being told their care was viewed as racist: “We go in with a rebuttal for those things. That’s our natural tendency. Like, I don’t think that’s true, here’s why.” Another participant observed, “No one wants to be called racist in front of their team. It’s not doing any good for anyone. It will probably end up in a defensive posture or response because you haven’t had time to think.” Participants wanted time to process difficult feedback in a safe space with their peers. One physician noted, “It might be helpful to have some time to debrief on that interaction and hear from your team.”

#### Receptiveness and Humility

Humility was repeatedly identified as necessary to having open and productive conversations about racism with patients: “It takes humility to realize that when patients are questioning, it’s not a challenge to your authority. It’s expressing discomfort.” Another stated, “What you want on the provider side is receptiveness and humility.” Participants thought it would be helpful to be reminded and prompted to focus on active listening, rather than responding: “Your first thing is maybe just to listen, anchor, and be curious.”

### Clinician Skills and Resources

#### Framework and Language

Many participants reported feeling they did not have the language or skills to discuss racism with their patients: “I am open to the idea that patients experience racialized healthcare and I would like to be able to have those conversations, but I’m afraid I’ll step on a landmine.” Participants wanted a framework and accompanying language: “Standard recommendations for these kinds of discussions, like language and framework. How do you have a discussion with someone who feels that you’re racist, between a privileged White person and a marginalized patient?” Participants thought patient advocates would be helpful, viewing them as a resource to guide them through their approach with one physician stating, “I want to be called out if I’ve communicated in some way that’s offending or noncommunicative.”

#### Objective Data on Subjective Experiences

Participants thought clinician-directed anti-racism trainings were important but limited in their ability to advance knowledge and skills in being an anti-racist clinician. Participants described themselves and their colleagues as data- and evidence-driven, identifying contextually relevant literature as helpful accompanying resources: “Everything is rational, scientific, and evidence-based.” Another remarked “You should provide data for people who are very scientifically minded. Then they may wonder, am I doing that too?” Another physician remarked, “I could imagine data to support self-reflection.”

#### Feedback to Medical Learners

Participants were concerned about racism perpetuated by residents and fellows and were uncertain how feedback to medical learners should be provided: “Residents and fellows are very vulnerable people.” Most strongly stated that they, as supervisors ultimately responsible for a patient’s care, should deliver feedback about racism to trainees: “I think it should be me.” Many participants wanted coaching and language to deliver this feedback: “You need to give attendings some skills.” Others voiced concerns about varying levels of skills among their peers in delivering difficult and hard-to-hear feedback: “I think you’re better off having a small set of people who are really good at that.” Another participant thought deidentified feedback to the entire team may be less distressing and broaden educational opportunities: “I wonder if there’s a way to talk to the whole team.”

### Supporting Patients

#### Empowerment

Many participants thought patients should have “lower-stakes” phrases to use when they were concerned about racism. One suggested a “set of questions if somebody felt they were receiving disparate health care.” For example, a Black physician suggested a phrase they use when asked a biased or racist question: “What is it about me that makes you ask that question?” Another Black participant drew upon personal experiences with racism and thought it was important to “have preventative tools going forward, strategies for future episodes, because they will happen again.” Similar to language provided to HCWs when patients use biased or racist language,^26^ participants thought a comparable approach for patients would be useful: “I have found those phrases to be helpful when I’m at a loss for words.” Participants recognized the added difficulty of navigating an unfamiliar system with its own values and norms, putting patients at a disadvantage to discuss racism. One participant stated, “A lawyer would never put their client on the stand without practicing. We don’t do that with patients. Just hope that works out for you.” However, participants were concerned about placing additional burden on Black patients to fix problematic interactions: “It puts the onus on the patient as opposed to clinicians to decrease their racist behavior.” Yet, many participants highlighted the importance of patients feeling empowered to discuss concerns about racism. One physician stated, “If the patient can have the language to tell the team what their concern is, it may be easier to open a conversation.” Another stated, “If a patient could feel empowered to say what they’re feeling, it would help even as we work to address some of our entrenched, structural problems.”

#### Safe Space and Advocates

Many participants noted they may not be viewed by patients as receptive and open-minded even if this was how they viewed themselves: “A patient would need to feel safe, that I would be empowered to help them or provide an empathic ear.” Many recognized medical settings as an unwelcoming environment where social status and privilege heavily favor clinicians: “The power dynamic when we’re talking to people in hospital gowns is just so apparent.” Participants identified lack of racial diversity among physicians as contributing to an environment that made patients feel unsafe. One participant noted, “It would be nice if our provider base was similar to our patient base. In our Division, we don’t have a single Black physician.” Similarly, participants noted the importance of a patient advocate like a family member or trusted clinician. One participant stated, “As a Black provider I can sometimes get more information from patients or they feel more comfortable talking to me.” Participants varied on whether the advocate should have a medical background when discussing racism. One physician stated that if they were non-medicalized, “the weird things we take for granted may land differently.” Another stated, “I think it’s best to have someone who’s been involved in the health care setting.”

Overall, these data suggest clinicians are open to discussing racism with Black patients with the support of additional resources suggesting a dual-facing program that concurrently involves both Black patients and clinicians, such as PRISM, may be helpful in addressing patient concerns around interpersonal anti-Black racism in the clinical setting.

## DISCUSSION

We described three themes identified by physicians as important to addressing concerns about interpersonal anti-Black racism. In general, participants expressed willingness to address concerns about racism with their patients but felt underequipped to do so. They were open to hearing concerns about care that was viewed as racist, even if they or a member of their team were the offenders. While participants felt Black patients should not be responsible for calling out racism, they were uncomfortable broaching the topic despite wanting to provide direct feedback to medical learners about anti-Black racism themselves. This raises uncomfortable and harmful tensions between vulnerable patients who may have multiple, intersecting axes of oppression and ill-equipped, emotionally unprepared clinicians. While discrimination, bias, and other forms of racism and its impacts are clearly well described, the ordinariness of racism and its everyday presence in clinical settings appears to be disappointingly underappreciated. Participants in our study unfortunately cited the need for data to increase their receptivity to patient concerns about racism despite numerous studies documenting the negative effects of racism in healthcare. This majoritarian approach, in which dominant narratives privilege physician perspectives and uphold white normativity, pathologizes and silences Black patients especially as they attempt to address concerns with physicians and other HCWs.^9,27,28^

A clinician component of a dual-facing program such as PRISM may comprise of ongoing bedside education about the everyday presence of racism as well as frameworks and resources for processing and responding to concerns about anti-Black racism. Some language has been proposed to help clinicians discuss racial inequities in care,^29^ but a paucity of literature exists in guiding clinicians through feelings of defensiveness and discomfort or the necessity of embracing humility and openness. Resources in other sectors provide some framework to help navigate discussing racism more broadly. For example, a five-part framework designed to help facilitate discussions about racism in the workplace highlights the importance of acknowledging uncomfortable realities and validating impacted persons’ experiences.^30^ Another framework emphasizes curiosity and acknowledging mistakes despite one’s intention to be anti-racist.^31^ These and other resources could be utilized in the clinical setting to support clinicians while allowing patients to be heard while promoting accountability.

Systemic racism is deeply entrenched in medical institutions as highlighted by the COVID-19 pandemic, the recent elimination of race from estimations of renal function, and the recent call to eliminate race and ethnicity from the interpretation of pulmonary function testing.^32–34^ There rightfully remains increased scrutiny over research, protocols, and policies that continue to systematically discriminate and perpetuate stigma against Black patients.^5,35,36^ While a dual-facing program such as PRISM may help hold some clinicians accountable for interpersonal anti-Black racism, systemic changes are still needed to actualize accountability on a larger scale. One method may be institutional uptake and implementation of restorative justice (RJ) conferences. RJ conferences are structured processes based on indigenous practices that allow community members to come together to address harmful behavior with a path toward accountability.^37^ RJ conferences have been used in multiple settings including schools, correctional and mental health facilities, and foster homes.^38–40^ RJ conferences focus on repairing harm inflicted upon impacted persons by allowing for a safe, mediated space for impacted persons to tell offenders how they were affected and deciding how offenders can repair the resulting harm. Offenders are given the opportunity to restore their standing in their community and repair relationships. As such, RJ conferences may provide one such space for patients and their families to share with HCWs how their actions affected them and have an active role in deciding how to hold offending HCWs accountable. RJ conferences create structured spaces for mending damaged relationships; fostering mutual respect; encouraging accountability, even if the resulting harm was unintentional; replacing feelings around revenge with moving toward racial healing; and reintegrating HCWs back into the clinical setting (5Rs).^37,41^ RJ conferences and similar processes may also help clinicians engage in race-conscious, reflective exercises to better understand patient-clinician dynamics within racialized medical settings as they reintegrate themselves back into clinical care.^42,43^

The elimination of health inequities ultimately requires moving away from deficit-based research approaches and processes that blames and silences Black patients and toward holding healthcare institutions and HCWs accountable.^44,45^ For example, standard research practices uphold white normativity by attributing health and healthcare inequities among Black patients with serious illness to their personal attributes rather than systemic factors of racist institutions.^6,7,44^ Indeed, while providing accountability on an interpersonal level addresses some of the concerns around pervasive practices that uphold white normativity and anti-Black racism, a redistribution of power away from healthcare institutions and toward patients who are standardly marginalized in racialized healthcare settings is ultimately needed. Critical research approaches to health inequities that account for anti-Black racism and center lived experiences that are discounted by research approaches that privilege dominant narratives and uphold white normativity, such as the Public Health Critical Race Praxis, work against invisible practices and thinking that continues to pathologize Black patients through a deficit-based research lens.^45–47^ Other frameworks and approaches to health inequities, such as Black feminist bioethics, center communities and move away from asking Black patients to discount their lived experiences and toward an approach that requires HCWs and institutions to first become trustworthy and accountable.^44,48^

### Limitations

There are limitations in our study. First, physicians who participated may have differing views from those who did not and schedules limited availability. Furthermore, participants with a working relationship with the interviewer may have been willing to be more open and forthcoming about their perspectives and experiences. Most participants were White, reflective of the physician population at the study site. Second, the study took place within a single urban academic medical center and results may not be transferable. Additionally, we did not include residents or fellows. Finally, the study design and interview scenarios were drawn from a related qualitative study of Black patients’ experiences, but patients were not directly included in this particular study.

## CONCLUSION

In this qualitative study, clinicians recognized the importance of hearing and discussing their patients’ concerns with racism in their care and identified both existing and additional resources for patients and clinicians to facilitate productive conversations. Both clinicians and patients should be actively engaged in the development and implementation of communication-based solutions to improve conversations about racism that prioritizes racial healing.

### Supplementary Information

Below is the link to the electronic supplementary material.

Supplementary file1 (DOCX 18 KB)
