# Supplementary material for: Physician Perspectives on Responding to Clinician-Perpetuated Interpersonal Racism Against Black Patients with Serious Illness
Source: J Gen Intern Med. 2023 Aug 24;39(11):1969–76. doi: 10.1007/s11606-023-08377-z (PMC11306464; doi:10.1007/s11606-023-08377-z)
Supplement: Supplementary file 1 — Supplementary file1 (DOCX 18 KB) [file 11606_2023_8377_MOESM1_ESM.docx]

**SUPPLEMENTAL MATERIAL**

**A. Serious illness diagnoses**

| **Supplemental Table 1. Diagnoses associated with a median expected survival of ≤2 years^10^** |
| --- |
| - Chronic Obstructive Pulmonary Disease with Forced Expiratory Volume in 1 second (FEV1) <35% predicted or oxygen dependence - Restrictive lung disease with Total Lung Capacity <50% predicted - New York Heart Association class III or IV heart failure - Left Ventricular Assist Device or Implantable Cardiac Defibrillator with age 65 years or older - Child's class C cirrhosis or Model for End-Stage Liver Disease>17 - Dialysis dependent renal failure and either diabetes or serum albumin <2.5 - Pulmonary Arterial Hypertension with 6 Minute Walk Test <250 feet - Cystic fibrosis FEV1<30% predicted - Aged 75 years or older with diagnosis of at least one of the life-limiting chronic illnesses noted above, although possibly of lesser severity - Hospitalization from any case within the pasts 18 months with diagnosis of at least 1 of the life-limiting, chronic illnesses noted above, although possibly of lesser severity - Aged 90 or older - Charlson Comorbidity Index score ≥6 |

**B. Interview Guide**

- What are your first impressions about PRISM? What do you like about this? What do you not like about this?
- Do you think there is something we could include that might help better address the distress of experiencing racism in clinical settings?
- From a provider perspective, if a patient wanted to discuss their experiences with racism and discrimination in the healthcare setting more broadly, what do you think would be the most helpful tools that would help them talk with you? What do you think they would need to know?
- Some of the feedback we have received from patients is that they also expect a clinician component to this program. What tool or skill would you like to have to help talk with a patient about racist healthcare experiences that involved you or your team.
  - What would make you feel prepared to support or engage with that patient?
  - What would make you or your team feel supported?

I’d like to present a scenario to you in which racism is an important contextual feature of an interaction between a clinician and a patient.

*Mr. Evans is a 64-year-old Black man with metastatic cancer. He is upset and feels that his pain is not being properly treated. He also thinks other treatment modalities are being withheld from him though he cannot say what exactly. He believes he is being mistreated because he is Black and doesn’t have a lot of money. He wants to talk to his doctors more about this, but every time he brings this up, he feels his treatment team is too quick to try to reassure him. Mr. Evans has recently completed the PRISM program and is ready to speak with his team more frankly about his medical treatment, including talking about whether or not race or money is playing a role in what the team decides to give him. He is worried about appearing as an angry Black man, especially since his treating team is made up of all White physicians.*

- What’s your first reaction when you hear this? What comes to mind?
- Have you been in a situation like this before?
  - How did you navigate it?
  - Was there anything that you wish you did differently?
  - What, if any, resources do you wish you had to help navigate this situation?
- How can the PRISM coach help you or other providers who might be in similar situations prepare for conversations like this?
- What are other resources might be helpful for providers in navigating such situations?
- Do you have any other thoughts about anything that we have discussed that you would like to tell me?
